# Determination of Moulting Events in Rock Lobsters from Pleopod Clipping

**DOI:** 10.1371/journal.pone.0074146

**Published:** 2013-08-23

**Authors:** Caleb Gardner, David J. Mills

**Affiliations:** 1 Institute for Marine and Antarctic Studies, University of Tasmania, Hobart, Tasmania, Australia; 2 WorldFish, Penang, Malaysia; 3 Australian Research Council Centre of Excellence on Coral Reef Studies, James Cook University, Townsville, Queensland, Australia; George Washington University, United States of America

## Abstract

Rock lobster growth is routinely measured for research to optimise management measures such as size limits and quotas. The process of estimating growth is complicated in crustaceans as growth only occurs when the animal moults. As data are typically collected by tag-recapture methods, the timing of moulting events can bias results. For example, if annual moulting events take place within a very short time-at-large after tagging, or if time-at-large is long and no moulting occurs. Classifying data into cases where moulting has / has not occurred during time-at-large can be required and can generally be determined by change in size between release and recapture. However, in old or slow growth individuals the moult increment can be too small to provide surety that moulting has occurred. A method that has been used since the 1970’s to determine moulting in rock lobsters involves clipping the distal portion of a pleopod so that any regeneration observed at recapture can be used as evidence of a moult. We examined the use of this method in both tank and long-duration field trials within a marine protected area, which provided access to large animals with smaller growth increments. Our results emphasised that determination of moulting by change in size was unreliable with larger lobsters and that pleopod clipping can assist in identifying moulting events. However, regeneration was an unreliable measure of moulting if clipping occurred less than three months before the moult.

## Introduction

Rock lobsters are Australia’s most valuable fisheries species group with the gross value of annual production in 2011 estimated at $AUD390 million [1]. The productivity of this valuable resource is strongly influenced by growth rates so considerable effort is directed to the collection of this information in fisheries research. Information on growth of lobsters is typically obtained through tagging studies where change in size is measured across a known time period. In many cases growth is treated as being continuous so that classical growth parameters can be fitted. This approach has been adopted successfully in some southern rock lobster ( *Jasus* *edwardsii* ) fisheries where time-at-large and the number of recaptures are sufficient to create a reasonable approximation of continuity, despite discontinuous growth caused by moulting [2].

In other cases, the discontinuous nature of lobster growth is a critical consideration and must be examined explicitly in tagging data A common situation is where both moulting and research sampling occur in a single annual pulse. This can lead to data sets where some tagged individuals recaptured after 12 months had not moulted, while others had undergone two moults [3]. McGarvey et al. [4] noted that in general, continuous growth approximations are inappropriate where intermoult periods are large and synchronised in relation to sampling. A first step in examining these issues in tagging data is establishing whether moulting has occurred between tagging and recapture events.

Establishing whether a crustacean has moulted between sampling events is generally straight-forward because a change in size is detected. However, where growth increments are so small as to be of a similar order to measurement error, or even negative [5], it can be impossible to establish if a moult has occurred. This problem in determination of moulting is not uncommon and occurs in SW Tasmania, Australia; the most important region for lobster harvests in Tasmanian waters [6,7]. Even in faster growth areas it can sometimes be difficult to determine whether moulting has occurred because damaged individuals tend to divert energy at moult to limb regeneration rather than to growth [8].

Establishing whether or not a moult has occurred in the interval between release and recapture of marked crustaceans may require the collection of information in addition to lobster size. Measurement of changes in the exoskeleton between moults has been undertaken through moult-staging of the cuticle [9,10], degree of carapace fouling [11], changes in radiometric isotope composition [12] or shell hardness [13,14].

Another option for assessing change through moulting is the clipping of a portion of an appendage, such as a pleopod, at the time of release and then determining if regeneration has occurred at recapture – regeneration is then taken to imply that a moult has occurred. Trimming of pleopods as part of Tasmanian lobster catch sampling commenced in the 1970’s as an alternative to dart tags for marking lobsters [15]. While T-bar tags are now the primary marking technique employed, pleopod clipping has been retained because it provides additional information on timing of moult. Over 40000 tagged lobsters have now been recaptured so there is a considerable database that could be used to refine information on timing of moulting. However, a complication is that if pleopods are clipped only shortly before moulting occurs, the pleopod will not regenerate. Here, we use data from both tank and field experiments to evaluate the use of pleopod trimming to determine moulting events.

## Methods

In all experiments, the pleopods of lobsters were trimmed with scissors to remove the distal half of the exopodite process as per routine fisheries tagging operations.

### Tank experiments

These experiments allowed us to determine the exact date of pleopod trimming and moulting. Lobsters were held in 1 m^3^ flow-through tanks and were fed every second day with whole mussels, chopped squid or pellets formulated for prawn culture. Tanks were covered with black plastic to reduce light and lobsters were provided with concrete blocks for shelter.

**Figure 1:**
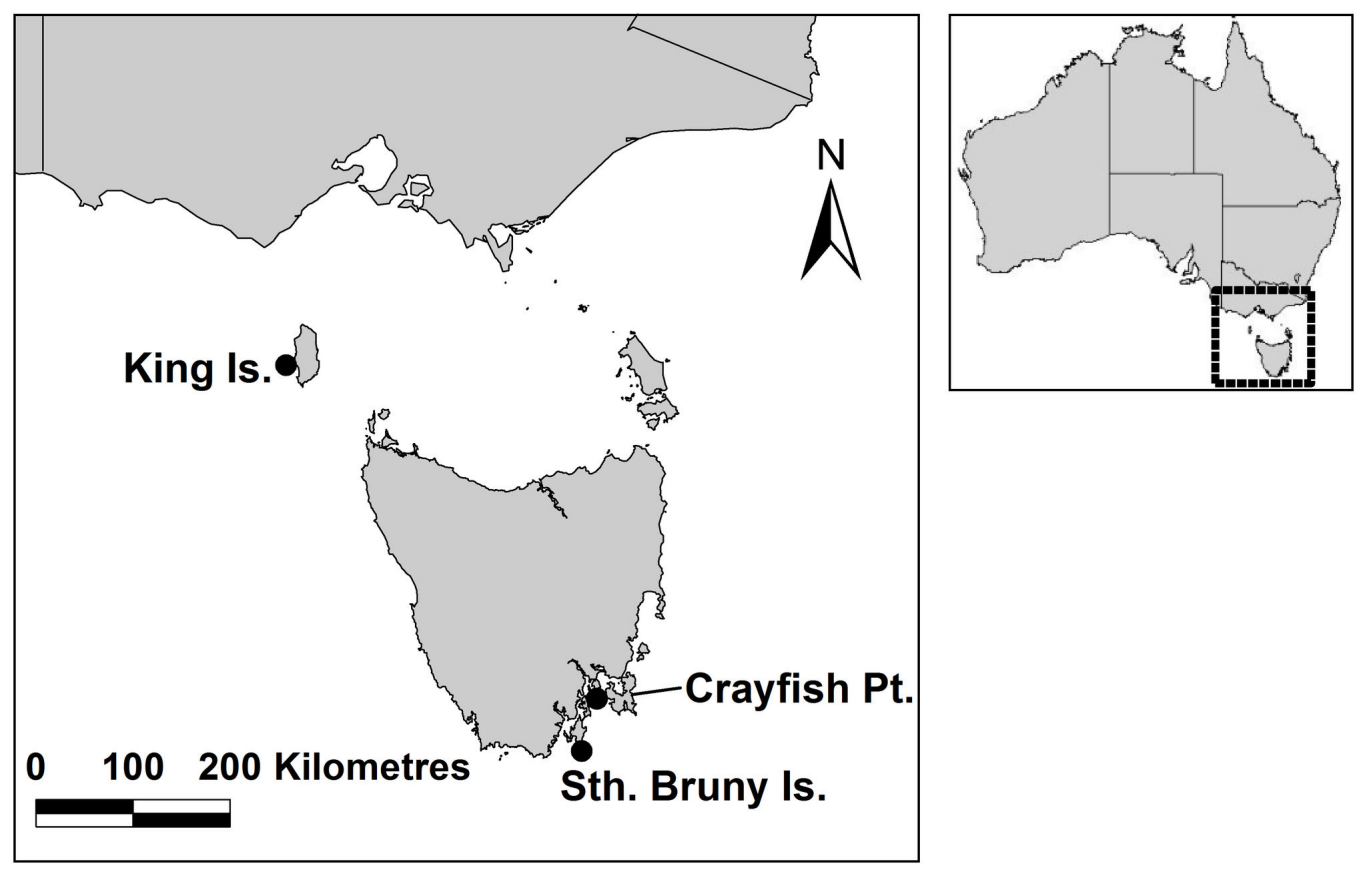
Sites of origin of lobsters for experiments. Lobsters from crayfish point were used for both field and tank trials while lobsters from the other sites were for tank trials only.

Male and mature female lobsters were obtained from three sites (King, Island, southern Bruny Island, and Crayfish Point; Figure 1). Growth rates vary dramatically between these regions [6] to the extent that female lobsters from southern Bruny Island almost never reach the size at first spawning of females from King, Island. As a result, it was not possible to include a similar size range of lobsters from each site. Given this constraint, we maintained at least 24 lobsters of each sex from each site broadly distributed across size ranges available from each region.

One of the eight pleopods was trimmed every two weeks. Animals were checked daily to determine the time of moulting and this date was used to back-calculate the days prior to moult that pleopod trimming occurred. Following moulting, the pleopods that had been trimmed were examined for signs of regeneration.

**Figure 2:**
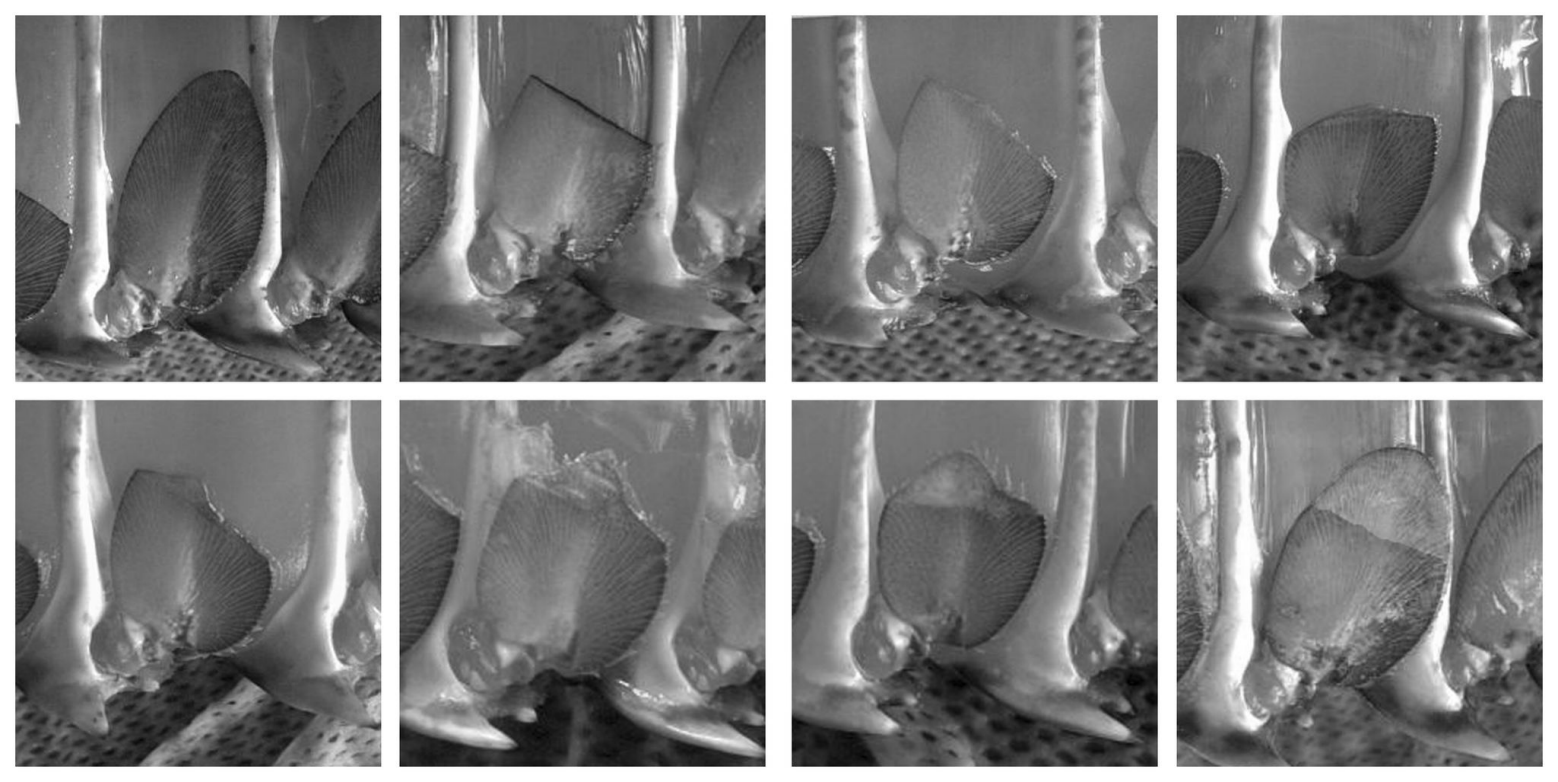
Categories of pleopod regeneration. A - untrimmed pleopod; B – no regeneration (NR); C to G – partial regeneration (PR); H – complete regeneration (CR). Regeneration in C and D is slight with greater risk of mistaken classification as “no regeneration” by staff in field trials. This subgroup of “partial regeneration” was categorised as “slight regeneration” in tank trials while the remainder were classified as “obvious regeneration”..

Post-moult pleopods were placed into one of three categories (Figure 2). The pleopods of animals with “obvious regeneration” had regrown so that it was clear that a moult had occurred. Any animal in this category would be detected by a brief glance from an observer during routine fisheries sampling. In other cases, regeneration was very slight with setae only visible along the trimmed margin, indicating that a new exoskeleton had been deposited. These were classed as “slight regeneration” and would only be apparent in field sampling by detailed inspection and could easily be missed. The final category was “no regeneration” where the post-moult pleopods had not even regenerated setae along the trimmed margin.

### Field experiments

Lobsters for the field experiment were collected by trapping in the Crayfish Point Reserve, which is closed to fishing. Permission to sample animals was provided by the Department of Primary, Industry, Parks, Water and Environment – Tasmanian Government (www.dpipwe.tas.gov.au). This approval was through a research permit covering the taking and holding of lobsters outside normal regulations. This study involved the collection of data in field studies but this did not involve threatened, endangered or protected species.

Sampling events were mainly conducted monthly although there were periods with longer intervals; a total of 43 sampling events were conducted within the 64-month period between August 1998 and November 2003. All lobsters captured that were greater than 80 mm CL were ventrally tagged in the second abdominal somite using individually marked t-bar tags (Hallprint Pty Ltd, 27 Jacobsen Crescent, Holden Hill, SA 5088, Australia). A total of 9785 lobsters were recaptured between sampling events including 5734 males and 4051 females. A further 348 recaptures occurred within the same sampling period as the release. As with tank trials, pleopods were trimmed with scissors at release and the extent of regeneration recorded on recapture. Pleopods on recapture were scored into three categories: no regeneration (NR), partial regeneration (PR) and complete regeneration (CR).

This area has relatively high growth so that the occurrence of a moult between recapture events was often established by change in carapace length. That is, change in size generally provided an indicator of moulting that was independent of pleopod regeneration data.

**Figure 3:**
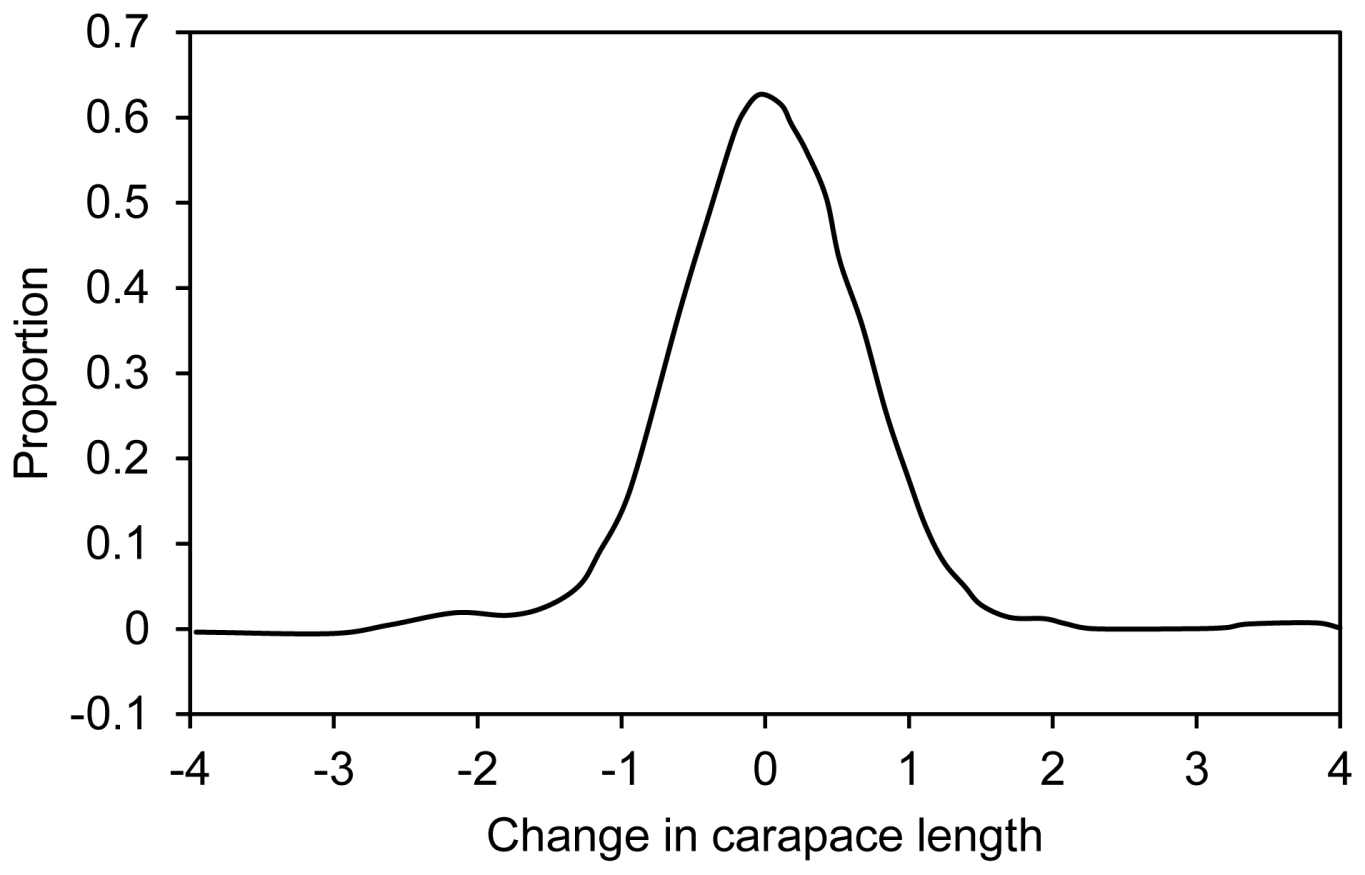
Error in measurements of length of lobsters sampled in field surveys. Change in length is the difference between release and recapture measurements for 348 recaptures obtained within 2 weeks of release.

Change in recorded size between release and recapture also occurred through measurement error. The 348 lobsters recaptured within two weeks of release provided data on measurement error, which was symmetrically distributed with 92.5% of lobsters measured to the precision of one mm and 96.6% measured to the precision of two mm (Figure 3). Recapture data were subsequently analysed in two ways: by treating changes in length of less than one mm and less than two mm as measurement error.

**Figure 4:**
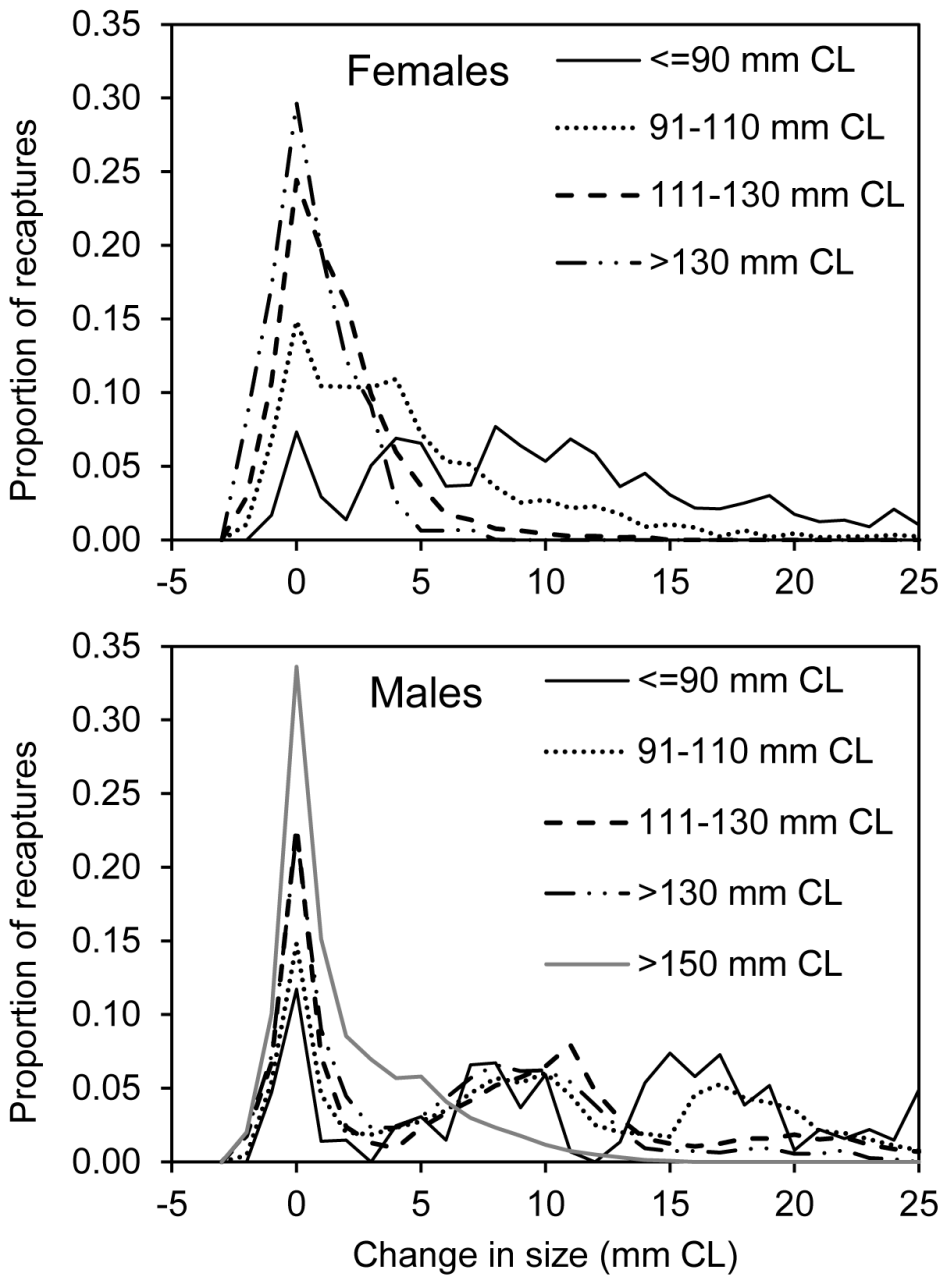
Change in size between release and recapture of tagged lobsters from Crayfish Point site.

Our ability to determine if a moult had occurred was diminished for larger lobsters because these animals underwent smaller moult increments, as expected with von-Bertalanffy type growth. Recapture data indicated that this problem was pronounced for female and male lobsters above 110 and 150 mm carapace length (CL) respectively (Figure 4). This was addressed by categorising release data into small and large lobsters separated by these thresholds of 110 and 150 mm CL.

### Data analyses

Recapture histories from trapping surveys were analysed to determine the timing of regeneration of pleopods and growth. The likelihoods of lobsters growing and regenerating pleopods in any given month were estimated by multistate Arnason-Schwartz (AS) tag/recapture models [16,17] run through Program MARK (http://www.phidot.org/software/mark/). These methods are a development of Cormack-Jolly-Seber (CJS) models [18–20] and provide a theoretical framework for estimating transition probabilities and associated errors.

Due to the data-intensive nature of this type of model, pleopod regeneration scores were compressed to 2 states – regenerated (including partial and complete regeneration) or not regenerated. Three possible state transitions were permitted in the model; from a state of no growth and no pleopod regeneration (NG-NR) as when first clipped, to states of no growth with regeneration (NG-R), growth and no regeneration (G-NR) or growth and regeneration (G-R).

Any lobsters that were likely to have moulted twice while at liberty (lobsters at liberty for more than 23 months) were removed from the data set. To further reduce the parameterisation of the model, recapture histories were compressed to a single 23 month period, with all lobsters considered to have been tagged within the first 12 months. While this precludes analysis of interannual variability in timing of moulting, it significantly boosts available data for each monthly cell within the model.

The fully parameterised (saturated) AS model can be represented by ϕ(ts) ρ(ts) ψ(ts). That is the likelihoods of survival (ϕ), resighting (ρ) and growth / regeneration transition (ψ) are a function of time of survey (t) and state (s). Reduced model structure was dictated by sampling and data constraints. All lobsters considered in this study survived through moult, so survival was constrained to equal 1. Resighting likelihood would be expected to vary with effort across all 23 sampling occasions, and was also allowed to vary between state (23 occasions x 4 states = 92 parameters). As we were interested in transition likelihoods on a monthly basis, transitions in like months within the 23 month period were constrained to be equal (i.e. the estimates for January in the 2 years would be the same). The resulting model had 124 parameters, of which two (survival =1 and transition likelihood for dis-allowed transitions = 0) were fixed.

## Results

### Tank experiments

**Figure 5:**
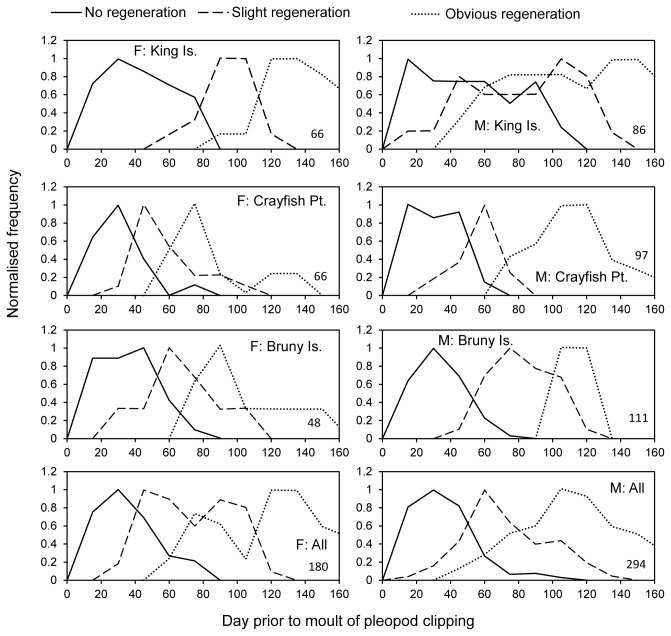
The extent of regeneration of pleopods from female (left column) and male (right column) lobsters. Pleopods were clipped at varying times prior to the moult and the extent of regeneration after moulting classified as: no regeneration (bold lines); slight regeneration (standard lines); or obvious regeneration (dashed lines). Upper pair of plots is for King, Island, second row is Crayfish Point, third row is South Bruny Island, and bottom row is all regions combined. Total sample size is given in bottom right of each plot. Time categories are in bins of 15 days and frequencies have been scaled to 1.

The extent of regeneration of pleopods increased with the time before moulting that trimming occurred (Figure 5). Both sex and site appeared to influence the rate of redevelopment of pleopods, although measurement of the effect of sex is confounded by the time of year the moulting occurs (Females in April/May, Males in September/October) and associated differences in water temperature. Site of origin of lobsters had a pronounced impact on regeneration even though lobsters were maintained in the same tanks for several months after capture. For both males and females, pleopod regeneration was most rapid in lobsters from Crayfish Point and slowest in lobsters from King, Island. Almost all pleopods trimmed longer than 60 days before the moult in lobsters from Crayfish Point showed signs of regeneration, although the pleopods of some King, Island animals failed to regenerate after 100 days. Obvious regeneration was apparent in most animals only after around 120 days, although this point was reached for Crayfish Point males at around 90 days.

### Field experiments

**Figure 6:**
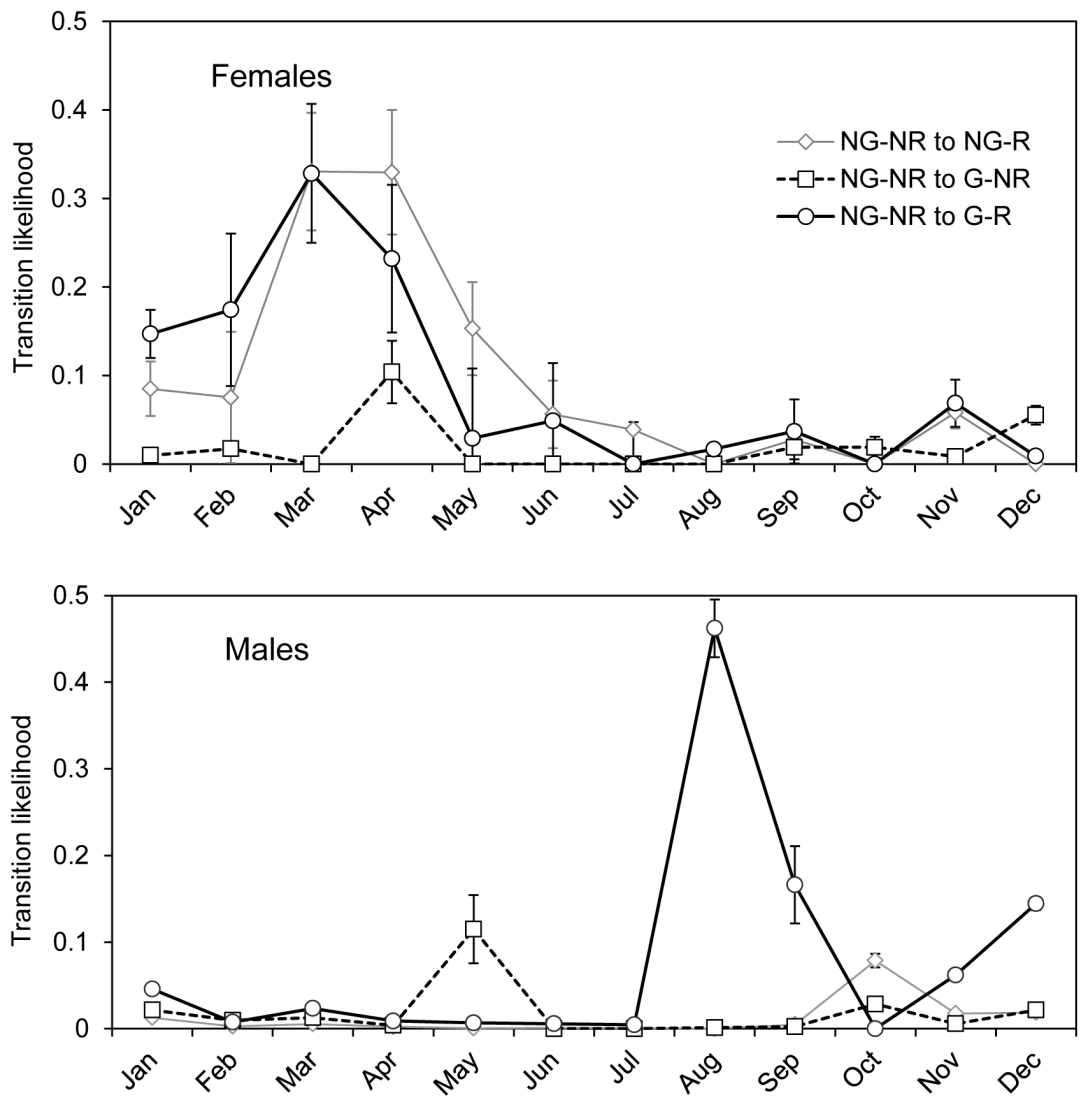
Determination of moulting from change in size and pleopod regeneration in a moderate growth area. Three categories of transition events are shown for each gender: (i) where size does not change but pleopod regeneration was observed, or from no growth / no regeneration to no growth / regeneration (NG-NR to NG-R); (ii) where size does change but pleopod regeneration was not detected, or from no growth / no regeneration to growth / no regeneration (NG-NR to G-NR); and (iii) where both change in size and pleopod regeneration was detected, or from no growth / no regeneration to growth / regeneration (NG-NR to G-R).

Analysis of data collected from field sampling indicated that moulting could almost always be detected by a change in size of male lobsters from this site, whereas change in size was a reliable indicator of moult of females in only about half of all cases (Figure 6). This result is site specific because growth traits and population structure vary between locations. In this case, the likelihood of detecting a female moult is presumably less than for faster growth areas or in populations with fewer older females. Nonetheless, the result shows that change in size can be inadequate for determining moult even at moderate growth sites. Female moulting was mainly spread across a four month period from February to May and was thus over a more protracted period than the male moult which occurred mainly in August and September. This result also emphasises the difficulty in moult determination of females because there is less clarity than for males on whether a tagged animal has transited across a moulting period.

There was a small likelihood of incorrect detection of moulting events for both males and females based on pleopod regeneration alone. This transition category peaked concurrently in April / May for both genders. This is not surprising in females because April is in the middle of the main moulting period, however, the observation was less expected for males. Even more surprising is that detection of moulting by males in May was only ever possible on the basis of change in size because pleopod regeneration was never detected for males in this period. The reason for this pattern is unclear although we speculate it may be caused by small males moulting twice per annum that were tagged and pleopod clipped only shortly before moulting. Regardless of the biological reason for this pattern, the conclusion for sampling design is clearly that detection of moulting in rock lobsters needs to utilise both change in size and pleopod regeneration data.

## Discussion

Measurement and modelling of rock lobster growth has been the subject of much research due to the value of rock lobster fisheries and the importance of growth information for quantitative fishery research. The use of von Bertalanffy curves and assumptions of continuous growth are widespread but can be problematic [21]. One of the common problems with this assumption in the case of lobsters is that recapture data used to estimate growth can contain animals with long time-at-large that have not moulted, or conversely, animals with very short time-at-large that have moulted. This potentially creates bias of parameter and variance estimates.

In an attempt to overcome biases of growth estimates, researchers often attempt to “clean” data to restrict records to those where moulting has occurred. Our results show that this can be difficult because moulting is not always well synchronised so it cannot be assumed that lobsters with time at large of 12 months have had an opportunity to moult once and only once. Further, change in size is not a reliable determinant of moulting and failed to detect moulting in this study for a large portion of tagged and recaptured female lobsters.

The regeneration of pleopods trimmed during catch-sampling clearly indicated that moulting has occurred since the previous release. However, an apparent lack of regeneration does not demonstrate that no moulting has occurred. This is because pleopods that are trimmed at a time close to the moult did not regenerate to any detectable degree. Even very fine scale regeneration of setae along the trimmed margin may not be detectable on pleopods trimmed almost 3 months before the moult. These results imply that pleopod trimming is only a reliable method for gauging whether moulting has occurred if the time-at-large exceeds 3 months and observers carefully scrutinise trimmed margins for setae. Where regenerated pleopods are only detected when regeneration is more obvious, then this time period would be longer, around 4 months.

The apparent effect of site of origin of lobsters is interesting given that animals were maintained in the same conditions for several months prior to the moult. The general trend was one of most rapid regeneration in lobsters from Crayfish Point, followed by southern Bruny Island, and slowest regeneration in lobsters from King, Island. This pattern doesn’t follow the actual cline in latitude and growth rates between the 3 sites. It’s possible that that the process of capture and transport to laboratory tanks impacted on the general health of animals so that animals transported greatest distance (King, Island, around 400 km) were more affected than animals transported shorter distances (Crayfish Point, around 10 km). If the health of animals in tanks was compromised, then the estimates presented here of time for pleopod regeneration to occur are likely to be overestimates of those in natural conditions.

In conclusion, we reiterate that change in size of tagged lobsters between release and recovery does not provide perfect guidance on whether moulting has occurred because increments can be smaller than measurement error. The clipping of pleopods at the time of first capture and collection of regeneration data on recapture can assist but this is also an imperfect determinant of moulting if the period between release and moulting is too short for regeneration to occur. Guidance on whether the moult is imminent and thus the ability to regenerate is low can potentially be gained from moult staging of the trimmed pleopod [10,22]. The implication for lobster growth research is that pleopod regeneration data provides valuable additional information on whether moulting has occurred and can identify moulting events not apparent from change in size data alone. This conclusion only holds where time between release and moulting is greater than 3 or 4 months, so for shorter periods some ambiguity about moulting events will remain.
